# Splenic artery aneurysm masquerading as upper gastrointestinal bleeding: A rare case report

**DOI:** 10.1016/j.ijscr.2025.110894

**Published:** 2025-01-15

**Authors:** Bishal Budha, Narayan Prasad Neupane, Bishweshwar Joshi, Dhiraj Poudel, Arjun Pandey, Rajan Budha

**Affiliations:** a Maharajgunj Medical Campus, Institute of Medicine, Tribhuvan University, Maharajgunj, Nepal; b Karnali Academy of Health Science, Jumla, Nepal

**Keywords:** Splenic artery, Celiac artery dissection, Aneurysms, Endovascular procedure, Mortality

## Abstract

**Introduction and importance:**

Splenic artery aneurysm is extremely rare but potentially life threatening disease which poses great challenge in diagnosing due to non-specific nature of clinical presentation. Rarely, it presents with upper gastrointestinal bleeding i.e. hematemesis and melena.

**Case presentation:**

A 58-years-old male presented with three and half month history of black tarry stool and abdominal pain, who was initially diagnosed as erosive gastritis and managed with antacids and PPI. After few months of resolution of symptoms, he experienced light-headedness, severe epigastric pain and syncopal episodes. That led to further imaging study which revealed splenic artery aneurysm with celiac artery dissection for which he underwent splenectomy after failure two repeat embolization intervention. Postoperative recovery was smooth, and he remained asymptomatic on follow-up.

**Clinical discussion:**

Though, there is constant risk of SAA to rupture, in our case pressure exerted by aneurysm on celiac artery caused dissection and upper GI bleeding. Endovascular technique is preferred technique but surgery reserved as options in case of failure.

**Conclusion:**

This case highlights the complexities in diagnosing and treating life threating splenic artery aneurysm with celiac artery dissection.

## Highlights

• SAA is rare but potentially life threatening condition, that occasionally manifest as upper gastrointestinal bleeding.
• Diagnosis of SAA is challenging due to its rarity an atypical presentation.
• Splenectomy is reserved as last resort method after failure of splenic artery embolization.

## 1 Introduction

Splenic artery aneurysm (SAA) is the potentially life threatening clinical entity which is defined as abnormal dilation of splenic artery >1 cm in diameter [1]. Although SAA is rare entity, it is the third most common visceral artery aneurysm, accounting 40–60 % following aneurysm of abdominal aorta and iliac artery [2,3]. SAA affect between 1 1000 to 1 in 2500 [4]. Histologically it is of two types: True SAA and Pseudo-SAA. Modifiable risk factors such as atherosclerosis, portal hypertension, trauma and chronic pancreatitis conditions contribute to SAA [5]. Small SAAs (≤2 cm) are asymptomatic and found incidentally during radiological interventions for other issues, whereas giant SAAs (≥5 cm) often present with vague symptoms leading ultimately to complications [1]. SAAs can be diagnosed radiologically and treated by open surgery, laparoscopic surgery, endovascular therapy, or a hybrid approach depending upon aneurysm's type, size, location and symptoms [5].

## 2 Case report

**Fig. 1:**
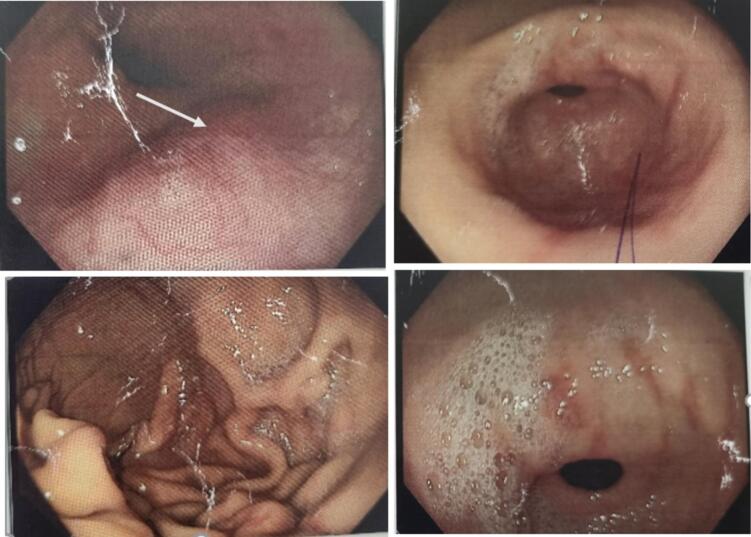
Upper-gastrointestinal endoscopy, showing normal fundus, normal D1 and D2 mucosa and multiple erosions in body/antrum (shown by white arrow).

**Fig. 2:**
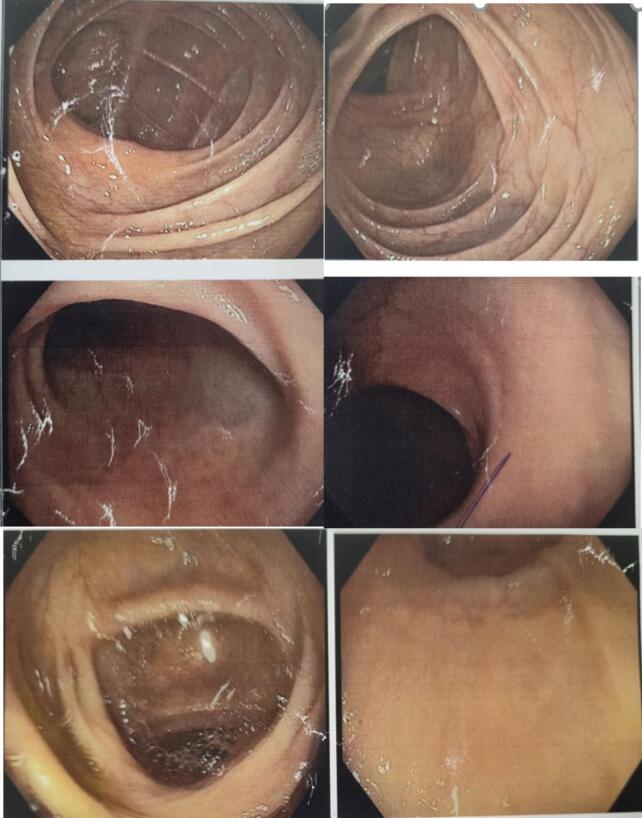
Ileo-colonoscopy showing normal colonic mucosa.

A 58-year-old, previously healthy male referred to our center, with three and half month history of back tarry stool and abdominal pain. Initially, patient experienced 2 to 3 episodes of black tarry stool per day over the course of three days, for which he was evaluated at local clinic with endoscopy and colonoscopy. After evaluating reports, she was diagnosed as erosive gastritis and was managed with antacids and proton pump inhibitors (PPI) (Fig. 1, Fig. 2). The patient remained asymptomatic for 2 months after which he experienced vague epigastric discomfort. Pain was intermittent type, relieved on its own, severe in intensity without radiation to other sites. The pain has no any relations with food intake and it was not relieved by medications. Other common causes of upper GI bleeding, such as peptic ulcer disease, esophageal varices, gastritis, Mallory-Weiss tear, or malignancies, are less likely as the patient lacks a history of chronic medication use, liver disease, coagulopathies, trauma, or other relevant risk factors. Prior to this patient has no history of trauma and drug intake other than antacids and PPI. Apart for this, he also gives history of light-headness and syncopal attack.

**Fig. 3:**
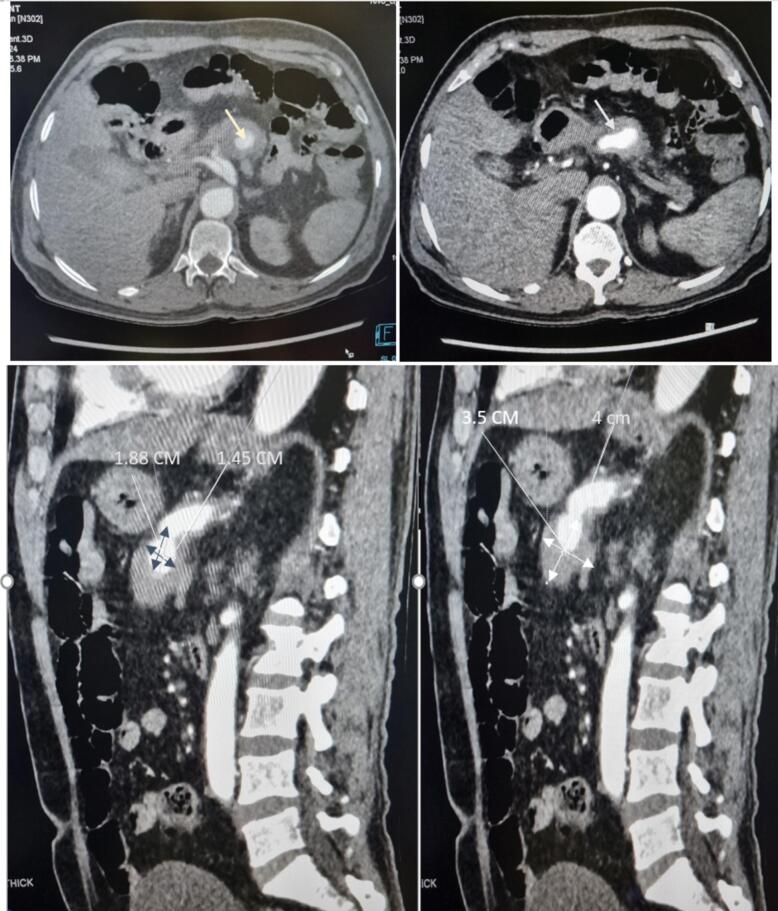
Contrast computed tomography showing outpouching of approximate 1.88 cm ∗ 1.45 cm and hypo-dense area of 3.5 cm ∗ 4 cm around outpouching.

**Fig. 4:**
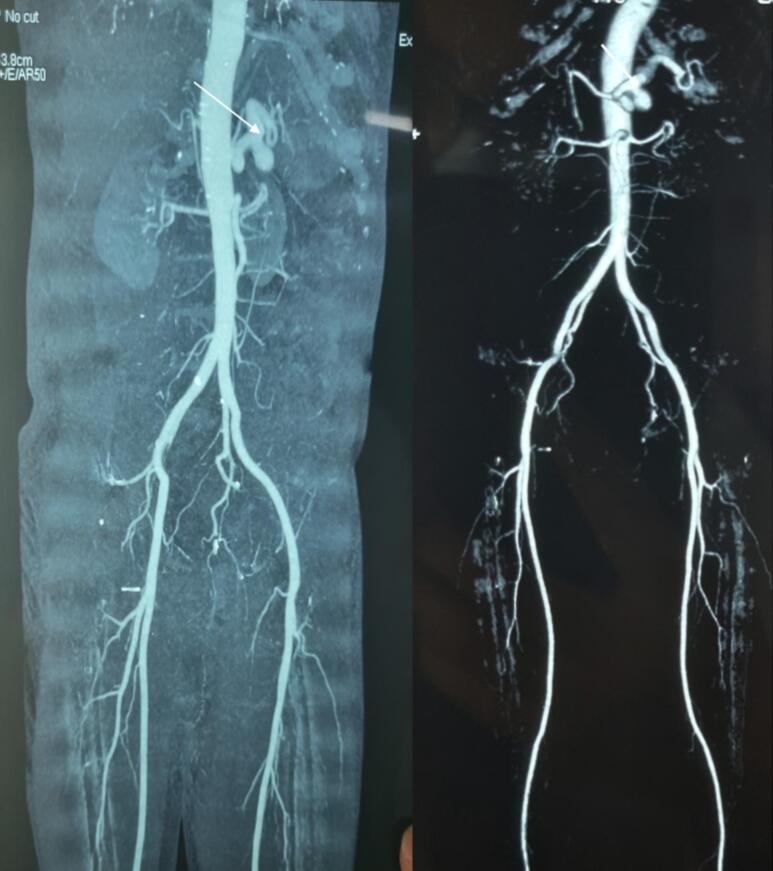
CT-angiography and 3D-reconstruction showing splenic artery aneurysm.

During his emergency visit, he was asked to do blood investigations and ultrasonography of abdomen. Blood reports revealed normal Aspartate aminotransferase (AST), Alanine transferase (ALT) and PT/INR but hemoglobin was 7 g% for which he was given 6 pints of blood. On USG, approximately 4 ∗ 4 cm size mass was present on pancreas, raising concern for further investigation. Then, he was asked to perform computed tomography (CT) of abdomen, which revealed 3.5 ∗ 4 cm size hematoma and extravasation of contrast into the adjacent bowel loop suggestive of enteric fistula (Fig. 3). To identify the source of bleeding, a CT angiography was done, which uncovered 1.88 ∗ 1.45 cm size splenic aneurysm (Fig. 3, Fig. 4). Furthermore, CT revealed isolated dissection of the celiac artery with extension of the dissection flap into splenic artery ostium, which suggest probable communication of the splenic artery from the false lumen with presence of large partially thrombosed aneurysm from its proximal segment.

**Fig. 5:**
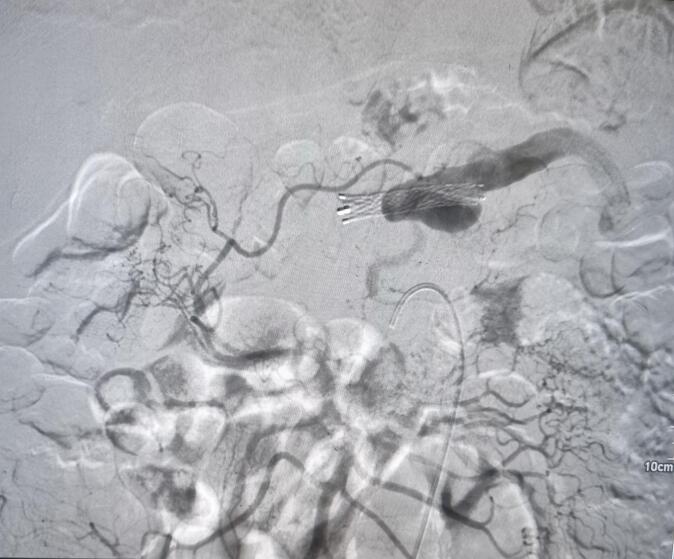
Image showing splenic artery embolization.

For this, he underwent splenic artery embolization and hepatic artery stenting, followed by close monitoring in medical ICU for five days. The ICU stay was uneventful and showed remarkable improvement. Upon discharge, he was advised to follow up in three months. However, just in 12 days of discharge, he once again experienced melena, hematemesis and severe epigastric pain. This necessitated a repeat embolization of splenic artery (Fig. 5). However, his symptoms didn't improve and he was planned for splenectomy. After being thoroughly informed about life after splenectomy, the patient received vaccination. With the consent of patient and splenectomy with ligation of splenic artery was done.

His postoperative recovery was smooth and without complications. He was discharged on 7th postoperative day. He is on regular follow up appointments and remains completely asymptomatic.

## 3 Discussion

Splenic artery aneurysm is the 3rd most common subtype of visceral artery aneurysm which is characterized by focal dilation of splenic artery by >50 % than normal vessel diameter [5]. This rare entity was first elaborated in 1770 by Beaussier in autopsies. Histopathologically, it is divided into 2 types: True SAA and Pseudo-SAA. True-SAA involves all fours layers of arterial wall while pseudo-SAA involves just one or two layers [4]. The incidence of SAA ranges from 0.09 % in autopsies to 0.78 % on arteriography which increases significantly to 10 % in elderly population [6].

SAA is a rare entity with relatively unknown etio-pathogenesis. Atherosclerosis, portal hypertension, abdominal trauma, chronic pancreatitis, liver transplantation, pregnancy, fibromuscular dysplasia or connective tissue disorder like Ehlers-Danlos syndrome and Marfan syndrome are considered as modifiable risk factors of SAA [5,7]. Peptic ulcer disease is the rarest cause of splenic artery aneurysm [8]. Elderly age and female gender are considered as non-modifiable risk factor of SAA. Pancreatitis and direct splenic artery trauma are considered as main causes of pseudo-SAA [8].

True-SAAs are remains asymptomatic unless they rupture or reach to a giant (≥5 cm) size whereas pseudo-SAA are often symptomatic because of their large size. Risk of rupture of true-SAAs is very low (2–3 %) whereas it is seriously high for pseudo-SAAs (37–47 %) leading to mortality in 90 % cases [9]. Only 5 % of patients becomes symptomatic and among them only 10 % present with upper gastrointestinal bleeding (hematochezia, hematemesis). Patient might experience vague abdominal symptoms like abdominal pain in epigastric or left upper quadrant region, sensation of fullness, nausea, vomiting or unexplained weight loss [5,8,10]. However, in severe cases, patient might present with hemodynamic instability and life threatening shock because of massive intraperitoneal bleeding after rupture of aneurysm. In our case, despite SAA not having ruptured, patient presented with bleeding manifestations. It was due to pressure exerted by aneurysm on coeliac artery leading to dissection and subsequently gastrointestinal bleeding.

The rarity and their nonspecific symptoms poses significant challenges in diagnosing SAAs. Though SAAs are hard to diagnose, Angiography followed by computed tomography (CT) and USG are widely performed for diagnosis [8]. Ultrasonography is the first choice diagnostic tool in pregnancy [1]. Digital subtraction angiography (DSA) is considered as gold standard for diagnosis and management as it allows trans-catheter embolization. Early diagnosis can prevent rupture of aneurysm and subsequent life-threatening hemodynamic instability and mortality. In our case, aneurysm was diagnosed after CT angiography.

There is always constant threat of spontaneous rupture of aneurysm, especially of pseudo-SAA. Therefore, timely treatment is crucial for both symptomatic as wells as asymptomatic SAAs. There are no well established guidelines for treatment. Mode of treatment completely depends upon severity of symptoms, age, sex, morphology, location of aneurysm and complications. Endovascular technique such as trans-catheter embolization, percutaneous injection, and endovascular stent graft are the first-line preferred treatment for SAA due to their lower morbidity [1,10,11]. The rationale for embolization before splenectomy is to achieve hemodynamic stability, minimize surgical risks, and facilitate safer surgery by reducing blood flow to the spleen, ultimately improving patient outcomes. However, open abdominal surgery remains the gold standard treatment, usually reserved for failure of endovascular interventions and complications. Surgical procedure includes splenic artery ligation, aneurysmectomy and splenectomy [11]. Our patient was managed with splenectomy after two unsuccessful splenic embolization intervention.

## 4 Conclusion

The rarity and atypical presentation like upper GI bleeding of SAA creates diagnosis difficulty leading to high mortality. Early diagnosis with imaging techniques and timely appropriate treatment with embolization or splenectomy are crucial for better prognosis and preventing severe complications.

## Author contribution

Dr. Bishal Budha: Writing, Original draft preparation, Editing, Study Concept.

Dr. Narayan Neupane: Editing, Study Concept.

Dr. Bishweshwar Joshi: Writing and original draft preparation.

Dr. Dhiraj Poudel: Manuscript reviewer.

Dr. Rajan Budha: Manuscript reviewer.

All authors have read and agreed to the final version of the manuscript.

## Consent

Written informed consent was obtained from the patient for publication of this case report and accompanying images. A copy of the written consent is available for review by the Editor-in-Chief of this journal on request.

The work has been reported in line with the SCARE criteria.

## Ethical approval

None.

## Guarantor

Dr. Bishal Budha.

## Research registration number

None.

## Provenance and peer review

No commissioned, externally peer reviewed.

## Funding

None.

## Conflict of interest statement

Authors have no conflict of interest to declare.
